# Supplementary material for: The effect of natural selection on the propagation of protein expression noise to bacterial growth
Source: PLoS Comput Biol. 2021 Jul 19;17(7):e1009208. doi: 10.1371/journal.pcbi.1009208 (PMC8321134; doi:10.1371/journal.pcbi.1009208)
Supplement: S1 Appendix — (PDF) [file pcbi.1009208.s001.pdf]

S1 Appendix for: The effect of natural selection on the propagation of protein expression noise to bacterial growth

Laurens H.J.Krah & Rutger Hermsen

S1 Appendix: Derivation of Eqs 2-8

Here we derive equations 2 and (therewith) 8 from the main text.

Growth rate as an intensive function of protein copy numbers

We assume that the growth rate μ is a deterministic, *intensive* function of the stochastic protein copy numbers $\mathbf{X}$. The intensivity of the growth rate implies that if all the copy numbers are multiplied by the same factor α, the growth rate does not change (*i.e.*, $\mu(\alpha\mathbf{X}) = \mu(\mathbf{X})$). Describing the growth rate as a function protein copy numbers is therefore equivalent to considering proteome mass fractions or concentrations. In our derivation, we start with a description based on copy numbers, but our final results will be in terms of proteome mass fractions.

We do not model the underlying causes of protein stochasticity, and instead assume the distribution of each protein species across a population is known. Protein copy numbers of individual cells thus differ, and because the growth rate of each individual cell is determined by protein copy numbers, also the growth rate differs between individuals cells. Writing the growth rate as a function of protein copy numbers moreover implicitly assumes that intrinsic fluctuations of all other cellular components (*e.g.*, nucleic acids, lipids, and metabolites) relax on timescales much faster than growth (seconds rather than minutes or hours), and their concentrations are thus determined by the protein copy numbers. Note that throughout the derivation, we do not consider any temporal dynamics of $\mathbf{X}$.

Linearisation of μ

Assuming that $\mathbf{X}$ is always close to some linearisation point $\widetilde{\mathbf{X}}$, we can write to first order:

$$\mu(\mathbf{X}) \approx \mu(\widetilde{\mathbf{X}}) \left(1 + \sum_i \left(\frac{X_i}{\mu}\frac{\partial \mu}{\partial X_i}\right)\bigg|_{\widetilde{\mathbf{X}}} \left(\frac{X_i - \widetilde{X_i}}{\widetilde{X_i}}\right)\right). \tag{S1.1}$$

In a snapshot of a population of cells, this linearisation then approximates the growth rate of individual cells given their stochastic vector $\mathbf{X}$.

Decomposition of CV_μ

When the noise in the copy numbers of different proteins is uncorrelated, it is straightforward to calculate CV_μ. First, using the definition of the Growth Control Coefficients of Eq 1 we can simplify equation S1.1 by setting $\widetilde{\mathbf{X}}$ equal to the mean expression levels across the population of cells, $\mathbb{E}[\mathbf{X}]$:

$$\mu(\mathbf{X}) \approx \mu(\mathbb{E}[\mathbf{X}]) \left(1 + \sum_i C_i^\mu \left(\frac{X_i - \mathbb{E}[X_i]}{\mathbb{E}[X_i]}\right)\right). \tag{S1.2}$$

Then, we use the basic properties of the variance, *i.e.*, for any two uncorrelated stochastic variables Y_1 and Y_2, and scalars a, b, $\mathrm{Var}[aY_1 + b + Y_2] = a^2\mathrm{Var}[Y_1] + \mathrm{Var}[Y_2]$, to write:

$$\mathrm{Var}[\mu] \approx \mu(\mathbb{E}[X])^2 \left(\sum_i (C_i^\mu)^2 \frac{\mathrm{Var}[X_i]}{\mathbb{E}[X_i]^2}\right). \tag{S1.3}$$

In the regime where Eq S1.1 holds, we can also read from Eq S1.2 that $\mathbb{E}[\mu] \approx \mu(\mathbb{E}[\mathbf{X}])$. This allows us to further simplify Eq S1.3:

$$\frac{\text{Var}[\mu]}{\mathbb{E}[\mu]^2} =: \text{CV}_\mu^2 \approx \sum_i (C_i^\mu)^2 \, \text{CV}_i^2, \tag{S1.4}$$

which is Eq 2. This is also a special case from the variance decomposition in [6].

As mentioned, all expectation values are based on population distributions (as opposed to the distribution of a cell lineage over time). This means that the mean growth rate also equals the population growth rate.

Optimisation

Next, we optimise the *mean* expression levels of the metabolic proteins to achieve the maximal *mean* growth rate. We assume that a fixed number of proteins has to be allocated for other things than growth (the H-sector proteins), and constrain the average total protein abundance $\sum_i \mathbb{E}[X_i]$ to a fixed value Ω. The optimisation can then be written as maximising $\mathbb{E}[\mu]$ over $\mathbb{E}[\mathbf{X}_{\notin H}]$, with the constraint $\sum_i \mathbb{E}[X_i] = \Omega$:

$$\underset{\mathbb{E}[\mathbf{X}_{\notin H}]}{\text{Max}} \left[\; \mathbb{E}[\mu] \; \middle| \; \sum_{i \notin H} \mathbb{E}[X_i] = \Omega - \mathbb{E}[X_H] \; \right].$$

To find the optimal genotype, we use the Lagrange Multiplier method, which in this case takes the form

$$\mathcal{L} = \mathbb{E}[\mu] - \lambda \left(\sum_{i \notin H} \mathbb{E}[X_i] - (\Omega - \mathbb{E}[X_H]) \right),$$

where we need to solve $\nabla \mathcal{L} = 0$. This gives:

$$\left. \frac{\partial \mathbb{E}[\mu]}{\partial \mathbb{E}[X_i]} \right|_{\mathbb{E}[\mathbf{X}]^*} = \lambda, \text{ for all } i \notin H, \tag{S1.5}$$

$$\sum_{i \notin H} \mathbb{E}[X_i]^* = \Omega - \mathbb{E}[X_H]. \tag{S1.6}$$

Here the asterisks indicate the optimal value of that variable. Using Eq S1.1 we can calculate $\mathbb{E}[\mu]$ explicitly:

$$\mathbb{E}[\mu] \approx \mu(\widetilde{\mathbf{X}}) \left(1 + \sum_i \left. \left(\frac{X_i}{\mu} \frac{\partial \mu}{\partial X_i} \right) \right|_{\widetilde{\mathbf{X}}} \frac{\mathbb{E}[X_i] - \widetilde{X}_i}{\widetilde{X}_i} \right),$$

which allows us to calculate the partial derivatives of Eq S1.5:

$$\frac{\partial \mathbb{E}[\mu]}{\partial \mathbb{E}[X_i]} = \mu(\widetilde{\mathbf{X}}) \left. \left(\frac{X_i}{\mu} \frac{\partial \mu}{\partial X_i} \right) \right|_{\widetilde{\mathbf{X}}} / \widetilde{X}_i, \text{ for all } i \notin H.$$

Next, we make a particular choice for the point of linearisation $\widetilde{\mathbf{X}}$ by setting it equal to optimal genotype, $\mathbb{E}[\mathbf{X}]^*$. In this vector, the partial derivatives equal λ (Eq S1.5):

$$\lambda = \left. \frac{\partial \mathbb{E}[\mu]}{\partial \mathbb{E}[X_i]} \right|_{\mathbb{E}[\mathbf{X}]^*} = \frac{\mu^* C_i^{\mu^*}}{\mathbb{E}[X_i]^*}. \tag{S1.7}$$

Here, the asterisk denotes that the equation is only valid at the vector with optimal mean expression levels. (Similar arguments were exploited in [37] and [38], although without the inclusion of the

H-sector and stochastic gene expression.) Lastly, we can use Eq S1.6, together with the sum rule for the GCCs (Eq 3), to calculate λ explicitly:

$$
\begin{aligned}
0 = \sum_i C_i^{\mu^*} &= \sum_{i \notin H} \frac{\lambda}{\mu^*} \mathbb{E}\left[X_i\right]^* + \sum_{i \in H} C_i^{\mu^*} \\
&= \frac{\lambda}{\mu^*} \left(\Omega - \mathbb{E}\left[X_H\right]\right) - \frac{\mathbb{E}\left[X_H\right]}{\Omega},
\end{aligned}
$$

and thus

$$
\lambda = \frac{\mu^* \mathbb{E}\left[X_H\right]}{\Omega(\Omega - \mathbb{E}\left[X_H\right])} = \frac{\phi_H}{\Omega(1 - \phi_H)}, \tag{S1.8}
$$

using to the definition of $\phi_i := \mathbb{E}\left[X_i\right] / \Omega$. Finally, by combining Eq S1.7 and S1.8 we find the Growth Control Coefficient for all proteins that do not belong to the H-sector in the optimal growth state:

$$
C_i^{\mu^*} = \frac{\phi_H}{(1 - \phi_H)} \phi_i^*. \tag{S1.9}
$$
