# Supplementary material for: The effect of natural selection on the propagation of protein expression noise to bacterial growth
Source: PLoS Comput Biol. 2021 Jul 19;17(7):e1009208. doi: 10.1371/journal.pcbi.1009208 (PMC8321134; doi:10.1371/journal.pcbi.1009208)
Supplement: S2 Appendix — (PDF) [file pcbi.1009208.s002.pdf]

S2 Appendix for:
The effect of natural selection on the propagation of protein expression noise to bacterial growth

Laurens H.J.Krah & Rutger Hermsen

S2 Appendix: Application to experimental data sets

In the main text we argue that, in optimised cells, proteins with a larger mass fraction obtain a larger GCC and noise contribution. To study what this entails in the context of realistic distributions of protein abundances, we applied this theory to two experimental data sets: Taniguchi *et al.* (reference [16] from the main text) and Schmidt *et al.* (reference [39] from the main text). The results are shown in Figs 1 and S1. We here provide details of these calculations.

The data set of Taniguchi *et al.*

The data set from Taniguchi *et al.* [16] contains a library essay of protein abundances (copy numbers) and copy number variances of over 1000 protein species of *E. coli*. We estimated protein mass fractions from this data set by assuming that they are similar to the relative copy numbers in the data set (*i.e.*, ϕ_i is estimated as the measured mean copy number of protein species i, divided by the sum of the mean copy numbers of all measured protein species, thus ignoring differences in molecular mass). Moreover, we assume that the measured expression noise in the different proteins is uncorrelated, even though extrinsic noise sources, which are responsible for the noise floor, are likely to add correlated noise to abundant protein species.

After estimating mass fractions, GCCs were set as follows. First, a fraction of ϕ_H of the proteome was randomly selected and assigned to the H-sector; their GCCs were set equal to minus their mass fractions (Eq 4). The other protein species were not part of the H-sector and thus assumed to be "metabolic" proteins. For those we compared three cases. Firstly, the proteins were assigned the GCCs calculated for optimal cells ($C_i^\mu \propto \phi_i$, Eq 5). Secondly, we considered the case in which all GCCs are equal. Thirdly, we took the GCCs of the optimal cells, but permuted them randomly, resulting in a cell in which the distribution of the GCCs is the same as in the optimal cell, but in which there is no relation between mass fraction and growth control. Next, for all three cases the noise contributions were calculated according to Eq 6. Results of the analysis are displayed in S1 Fig.

We moreover analysed the effect of ignoring the noise floor by setting protein variances to $CV^2 = F/\mathbb{E}[X]$ ($F = 2$). As expected, a noise floor causes abundant protein species to relatively contribute more strongly (compare Fig 1, with a noise floor, to S1A Fig, without). Indeed, the noise floor mostly affects the CV of abundant protein species. However, the noise floor also strongly decreases the (relative) noise contribution of low-copy-number proteins (S1B Fig).

The data set of Schmidt *et al.*

The extensive data set from Schmidt *et al.* [39] contains measured protein abundances (copy numbers) of over 2000 *E. coli* protein species, under various growth conditions. Since also the molecular mass of each protein species was given, we included these in our calculation of mass fractions. We further analysed abundances measured during growth on glucose, a growth medium for which the overall expression level of catabolic proteins is experimentally shown to be optimal [24]. Since variance in expression levels was not measured, we set the CV for each protein species according to Eq 7, but added a noise floor to each protein species to mimic observed variances in Taniguchi *et al.*, i.e., $\mathrm{CV}_i^2 = F/\mathbb{E}[X_i] + n_f$, with $F = 2, n_f = 0.15$. Lastly, the same calculations were performed as described above for the data set of Taniguchi *et al.* The results are displayed in S1C Fig.

Note that due to the many assumptions, the noise contributions are only rough estimates, and should not be considered quantitative predictions.
