# Supplementary material for: The effect of natural selection on the propagation of protein expression noise to bacterial growth
Source: PLoS Comput Biol. 2021 Jul 19;17(7):e1009208. doi: 10.1371/journal.pcbi.1009208 (PMC8321134; doi:10.1371/journal.pcbi.1009208)
Supplement: S1 Table — Catabolic rate (kcat) and inhibition constant (kinhi) for the five metabolic protein species in the kinotype used to create figure Figs 2 and S4. Additionally, the initially sampled (relative) protein abundances are given. (PDF) [file pcbi.1009208.s009.pdf]

S1 Table for:
The effect of natural selection on the propagation of protein expression noise to bacterial growth

Laurens H.J.Krah & Rutger Hermsen

S1 Table: Kinetic parameters of the example kinotype

Table S1. Kinetic parameters; k_{cat} and inhibition constant (k_{inhi}) for the five metabolic protein species in the kinotype used to create Figs 2 and S4. Additionally, the initially sampled (relative) protein abundances are given.

Protein	k_{cat}	k_{inhi}	ϕ_{initial}
1	3.4048	0.2153	0.19177
2	4.3489	4.3831	0.0080544
3	1.8454	3.6435	0.18137
4	3.1650	6.5335	0.084103
5	5.4577	1.8425	0.1347
