# Supplementary material for: The effect of natural selection on the propagation of protein expression noise to bacterial growth
Source: PLoS Comput Biol. 2021 Jul 19;17(7):e1009208. doi: 10.1371/journal.pcbi.1009208 (PMC8321134; doi:10.1371/journal.pcbi.1009208)
Supplement: Attachment — Submitted filename: Response to Reviewers.docx [file pcbi.1009208.s010.docx]

**Response to Reviewers**

*First of all, we would like to thank the reviewers for their thoughtful comments and suggestions. Based on their remarks, the manuscript has significantly improved. Most importantly, we have now rewritten the introduction to provide more context to the study, made several changes to avoid confusion, and added an additional analysis (including figures 1, S1 and Appendix S2) in order to evaluate the possible impact of the effect proposed in this study.*

*Below, we answer the questions of the reviewers in a point-by-point fashion.*

___________________________________________________________________________

**Reviewer #1:** A well written and interesting manuscript. Some specific comments:

L 70: It might be worst stating explicitly that the expectation is defined over a distribution for X (which, in turn, is defined in terms of a population of cells)

*Thank you for this suggestion. We agree that this is a crucial point and now emphasize this both in the main text and in Appendix S1.*

L 161: please include a bit more detail for the derivation of equation 7. I’m not reaching this conclusion by combining 4,5, and 6 (and using the definition on line 117). It strikes me that we should get different results depending on whether i is in H or not (in which case I presume this is the "i not in H" case.

*We recognize that the derivation was not clear and that the distinction between proteins that belong to H and those that do not should have been made explicitly. We adjusted the text to clarify.*

Figure 2A: are there meant to be two dashed lines in this figure? and red dots along the H-protein line; are some of the dots H-proteins?

*We thank the reviewer for this sharp observation. One of the dashed lines had been removed by error; we placed it back.*

*None of the proteins here are H-sector proteins. But if other proteins are over-expressed their GCC also becomes negative. Moreover, if they are sufficiently overexpressed their GCC converges to the result for the H-sector proteins. This can be understood as follows.*

*The prediction for the H-proteins essentially results from the realisation that the main effect on the growth rate of an investment in H proteins is that this investment goes at the expense of other enzymes that could have increased the metabolic flux and hence the growth rate.  The same holds for metabolic proteins that are already expressed in excess: any further investment in their expression affects the growth rate mainly through the concomitant reduced investment in other enzymes. We made adjustments to the text to highlight this: lines 236-237 and 247.*

L326: suggestion: can the function of operons be understood in this context? (*i.e.* not just coordinated expression but also, to an extent, resulting concerted variance in abundance)? Perhaps this case can be treated trivially by lumping all enzymes coded by an operon into a single ‘meta-protein’ to which your analysis applies directly. Just a thought.

*Yes, since the expression levels of proteins that are coded in the same operon are correlated, they can be modelled as a single unit, although the mean expression of each protein has to be optimised separately to arrive at the “optimal” genome.  Inspired by this reviewer's comment we have amended our statement about the “meta-modelling” approach, a possible way of dealing with correlated expression noise.*

*Incidentally, earlier theoretical work has indeed hypothesised that one function of operons could be to ensure that the noise in the expression of interacting proteins becomes correlated  (Sneppen et al., 2010)*

L 351: It may be other referencing here the work by Terry Hwa’s group on protein partitioning and its relation to growth rate and other environmental factors, e.g.. Klumpp, Stefan, and Terence Hwa. "Bacterial growth: global effects on gene expression, growth feedback and proteome partition." Current opinion in biotechnology 28 (2014): 96-102.

*At the specified location in the manuscript, we now include a reference (from Hwa and others) that describes an FBA-like technique that also makes use of the proteome partitioning framework.*

Appendix S1: cell mass is described in terms of X (protein only). There’s I suppose, an unstated assumption that other contributors to cell mass (lips, nucleic acids), are proportional? (protein constituting maybe about 55% of the cell mass <http://book.bionumbers.org/what-is-the-macromolecular-composition-of-the-cell/#:~:text=So%20the%20lipid%20bilayer%20occupies,this%20value%20to%20%E2%89%8814%25>.)

*Our assumption is that the growth rate is a sole function of protein copy numbers. This does not necessarily imply that the other contributors to cell mass are proportional. Instead, we envision that all other concentrations (lipids, nucleic acids, metabolites) are also determined by the protein copy numbers through (unmodelled) metabolic reactions that equilibrate rapidly. We made these assumptions more explicit at the start of S1. Moreover, we now introduce Omega not as the total cell mass, but as the total protein abundance (see also statement below).*

Appendix S2: ’the flux through *each of* the first four steps is given by…
*Agreed; we have adjusted this sentence.*

Omega can perhaps be defined more specifically as mean total protein abundance.

*Agreed, and we  now introduce it as such.*

*___________________________________________________________________________*

**Reviewer #2:** I enjoyed reading this work. I think the main result is of interest to a general audience, however little effort is made to make the text understandable, and of interest, to a more general audience than the biophysicist, familiar with key single-cell noise results.

*We would like to thank the reviewer for his nice words and constructive criticism. To improve the appeal of the manuscript to a broader audience, we now include a longer, more general introduction, and have included an additional analysis that demonstrates the potential implications of the proposed effect (figures 1, S1).*

Also how significant this effect really is, when one consider for instance realistic *E. coli* numbers and key abundant enzymes, such as the ribosome or glyceraldehyde-3-phosphate dehydrogenase, is unclear and would help the reader in appreciating the importance of the result in this work.

*We agree that an example application to real data can give a good indication of the effect size and help interpretation from readers that are less interested in the details of the theory. Therefore, we added to the result section an application of the theory to a data set of E. coli protein abundances (and expression noise levels), and also added in Appendix S2 an application to a second data set.*

I also miss a discussion of the relevance of protein copy number vs concentrations, isn't concentration noise the relevant measure for this purpose?

*We agree with the reviewer that, commonly, noise in protein concentrations is considered. However, throughout the article we consider the growth rate as an intensive function (i.e. scaling all protein   copy numbers with a constant, for example volume, does not change the growth rate). In our derivation, we therefore start with protein copy numbers for ease of interpretation and notation. Note that our final results are in terms of mass-fractions (assumed proportional to concentrations). To make this more explicit, we added a section at the start of Appendix S1.*

Finally, noise in abundant protein is likely very small, in particular when noise in concentrations is considered, but how small? And whether the effect emphasised by the authors is truly significant in realistic situations remains unclear and deserves more attention for this work to be of interest to a more general audience than biophysicists developing novel theories -- I see this more as an intermediate result on the way to something more profound than a final result*.*

*Again, we agree with the reviewer. As we mentioned above, we now include a new analysis to demonstrate the significance of the effect in the context of realistic protein abundances and noise levels for E. coli, as measured and published in earlier publications.  Hopefully, this gives the reader a clear impression of the amplitude of the noise in abundant proteins. In addition, the analysis demonstrates that natural selection on the growth rate can result in a truly significant shift in the distribution of noise contributions among proteins.*

*___________________________________________________________________________*

**Reviewer #3:** The manuscript by Krah and Hermsen investigates the propagation of protein noise to noise in bacterial growth. This is an interesting and timely manuscript. The authors built on their earlier work (ref 19) that introduces a sum rule for growth control coefficients and show that in cells that have optimised their growth (through natural selection), counter intuitively, the highly expressed genes (that have lower intrinsic noise) have the largest contribution to the growth rate variability. The authors show this in an idealised case, analytically and through simulation of a specific stochastic toy model. I have the following specific comments on the paper.

*We thank the reviewer for the kind words.*

The toy model is not fully stochastic. They assume expressions are static and follow a gamma distribution, but they are fixed and obtain the growth rate by solving systems of ODEs. Why did the authors not consider a fully stochastic model (using Gillespie simulations, for example).

*In the whole manuscript (including the Toy Model), we assume that gene expression is stochastic. For convenience, we do not model gene expression explicitly as a dynamical process, but instead take the resulting probability distribution of X in a population of cells as our starting point. Provided the growth rate μ can be considered a function of the expression levels of the proteins, this is enough for our purposes, because the distribution of X then directly determines the distribution of μ, so that the precise dynamics of X that result in the distribution become irrelevant.*

*In the Toy Model, the function μ(X) emerges from an explicit (albeit highly simplified) model of a metabolic network. The reviewer is correct that this metabolic network is not modeled stochastically, but rather as a set of ODEs.  The implicit assumption behind this choice is a separation of time scales: The fluctuations in the protein copy numbers are assumed to be much slower (hours) than the equilibration time of the metabolic network (seconds). As a result, the growth rate at a given phenotype X can be determined by calculating the equilibrium state of the ODEs describing metabolism. Also, because metabolism is fast, the stochasticity of individual metabolic reactions is averaged out over the time scales of the fluctuations of protein expression.*

*The assumption of time scale separation has now been made explicit in Appendices 1 and 3, and in the main text we have further clarified that no temporal dynamics are considered.*

I am also confused with the so-called mutations and low and high noise regime, in their toy model. I believe they are simply considering two situations, one where growth is optimal (using a numerical optimisation of concentrations of the proteins, in the low noise case) and one where growth is not optimal and parameters are random (the high noise case, although, they still say “a mutant genotype is accepted if it yields a higher growth rate”).

*We apologize for the confusion and have clarified the text in the hope to better explain our simulation. We distinguish two regimes: the small-noise (low variance in protein expression) and the high-noise regime (large variance). In the small-noise regime, the optimal gene expression pattern can be determined very accurately using a gradient-ascent algorithm because the mean growth rate E[μ(X)] is approximately equal to the growth rate in the mean expression pattern μ[E[X]]. In the high-noise regime this is not feasible because in this regime determining the mean growth rate E[μ(X)] requires sampling from the high-dimensional probability distribution P(X). Therefore, gene expression is evolved via a (Monte-Carlo-like) evolutionary algorithm: mutations are introduced, and these mutations are accepted if they yield a higher mean growth rate). We find that depending on the noise level, different mean expression levels result in the highest growth rate, i.e. which genotype is optimal depends on the amplitudes of gene expression noise.*

*We now tried to formulate the distinction between the two regimes more clearly.*

They also use some arbitrary number of repeats (15). I am very confused about the significance of the specific choices and the generality of the results. I find invoking the idea of mutations a bit confusing, could the authors not explore two situations (one where X’s are optimal for growth and one where X’s are not optimal for growth).

*We repeated each evolutionary stochastic simulation multiple times (15) to avoid the possibility that simulations become trapped in a local optimum. The number of repeats is indeed somewhat arbitrary; we chose it large enough to be confident that our results are replicable, but low enough such that the computation remains feasible.*

I have a related point of confusion; the authors look at the effect of noise in expression in the optimal growth state on variability of growth rate. However, gene expression variability could have a detrimental effect on growth rate itself (for example variability in expression of an essential low expressed enzyme could halt growth). Again, as the authors do not consider a fully stochastic model, they cannot address this issue.

*We suspect that this question is related to the question above, but also to the distinction between (1) the effect of expression noise on the variance* *of the growth rate and (2) the effect of expression noise on the mean* *growth rate.  To reiterate: our analysis does treat the expression of the proteins as a stochastic process (although there is no need to explicitly simulate its dynamics in time for our purposes). Moreover, the (possibly detrimental) effect of expression variability on the mean growth rate is certainly taken into account. In fact, both in our derivation as in the Toy Model the mean growth rate is optimised. In the Toy Model, we explicitly include in the calculation of this mean also those states in which the growth rate is strongly hampered by an enzyme that is lowly expressed  due to stochasticity. We refer the reviewer to what is now figure S2B, where we show that many slow-growing phenotypes are indeed caused by an essential, too lowly expressed protein. (An issue that evolution adequately addresses as shown in Fig S2D).*

*We have tried to avoid further confusion by explaining the Toy Model in more detail and making more explicit how the mean growth rate is calculated and optimised by the evolution simulation (Appendix S3).*

The authors do not consider extrinsic noise, but they should explain what happens if a constant baseline as extrinsic noise is considered in their analysis.

*We thank the reviewer for this good suggestion. We added such an analysis in the supplementary text Appendix S2, where we also estimate noise contributions from an experimental data set.*
